# New Evidence Confirms That the Mitochondrial Bottleneck Is Generated without Reduction of Mitochondrial DNA Content in Early Primordial Germ Cells of Mice

**DOI:** 10.1371/journal.pgen.1000756

**Published:** 2009-12-04

**Authors:** Liqin Cao, Hiroshi Shitara, Michihiko Sugimoto, Jun-Ichi Hayashi, Kuniya Abe, Hiromichi Yonekawa

**Affiliations:** 1 Laboratory of Mouse Models for Human Heritable Diseases, The Tokyo Metropolitan Institute of Medical Science (Rinshoken), Tokyo, Japan; 2 Technology and Development Team for Mammalian Cellular Dynamics, BioResource Center (BRC) RIKEN Tsukuba Institute, Ibaraki, Japan; 3 Graduate School of Life and Environmental Sciences, University of Tsukuba, Ibaraki, Japan; Karolinska Institutet, Sweden

## Abstract

In mammals, observations of rapid shifts in mitochondrial DNA (mtDNA) variants between generations have led to the creation of the bottleneck theory for the transmission of mtDNA. The bottleneck could be attributed to a marked decline of mtDNA content in germ cells giving rise to the next generation, to a small effective number of mtDNA segregation units resulting from homoplasmic nucleoids rather than the single mtDNA molecule serving as the units of segregation, or to the selective transmission of a subgroup of the mtDNA population to the progeny. We have previously determined mtDNA copy number in single germ cells and shown that the bottleneck occurs without the reduction in germline mtDNA content. Recently one study suggested that the bottleneck is driven by a remarkable decline of mtDNA copies in early primordial germ cells (PGCs), while another study reported that the mtDNA genetic bottleneck results from replication of a subpopulation of the mtDNA genome during postnatal oocyte maturation and not during embryonic oogenesis, despite a detected a reduction in mtDNA content in early PGCs. To clarify these contradictory results, we examined the mtDNA copy number in PGCs isolated from transgenic mice expressing fluorescent proteins specifically in PGCs as in the aforementioned two other studies. We provide clear evidence to confirm that no remarkable reduction in mtDNA content occurs in PGCs and reinforce that the bottleneck is generated without reduction of mtDNA content in germ cells.

## Author Summary

Mutations of mtDNA are responsible for many types of mitochondrial diseases in humans, including myopathy and neurological disorders. Females carrying a mixture of mutant and wild-type mtDNA variants transmit a variable amount of mutant mtDNA to each offspring. The proportion of mutated mtDNA inherited from the mother determines the onset and severity of diseases. Studies have suggested that the mtDNA genome is transmitted through a bottleneck, but the underlying mechanism remains controversial. By detecting mtDNA copy number in single cells, we previously showed that the bottleneck occurs without reduction of mtDNA content in germline cells. However, recently a study reported a marked decline of mtDNA copies in embryonic germ cells and attributed this reduction to the creation of the bottleneck. Yet another study concluded that the bottleneck occurs during postnatal oocyte maturation and not during embryonic oogenesis. To resolve these controversies, we examined mtDNA copies in embryonic germ cells identified using the same methodology as in the other two studies. We show solid evidence to confirm our previous findings. This confirmation is important because the understanding of mtDNA content in female germ cells will facilitate the development of therapeutic strategies preventing the transmission of mitochondrial diseases from mother to offspring.

## Introduction

Mammalian mitochondrial genome shows a 5 to 10 times greater mutation rate than the nuclear genome [1],[2]. This elevated mutation rate coupled with clonal maternal transmission leads to the high mtDNA polymorphism in populations. However, despite the prevalence of genetic variance within a species, most individuals possess only a single mtDNA variant. Pedigree analyses of heteroplasmic individuals in cattle, mice and humans revealed that mtDNA genotypes shift rapidly among offspring and return to homoplasmy in some progeny within a few generations [3]–[8], suggesting that a mtDNA bottleneck accounts for the rapid segregation. Early studies have proposed that the bottleneck occurs in embryonic development in consequence of a drastic reduction of mtDNA content in PGCs [9],[10]. In mice, the size of the bottleneck is estimated as to be ∼200 mtDNA segregation units [11].

To test these hypotheses, three independent research groups have attempted to quantify mtDNA copy number in single germ cells at different developmental stages in mice. Cao et al. [12] made the first direct measurements of mtDNA copy number in single PGCs (identified by endogenous alkaline phosphatase (ALP) activity) in wild-type mice using quantitative real-time PCR (qrt-PCR) and found that PGCs contained consistent amounts of mtDNA with a mean of ∼1350–3600 copies per cell between 7.5 days post coitum (dpc) and 13.5 dpc, indicating that the bottleneck occurs without a marked reduction of mtDNA copies in PGCs. Recently using *Stella*-GFP transgenic mice to isolate PGCs, a study determined a mean of ∼450 mtDNA copies per PGC at 7.5 dpc (median ∼200) and a mean of ∼1100–2200 copies between 8.5 dpc and 14.5 dpc. The drastic reduction in PGC mtDNA content at 7.5 dpc was suggested to be the cause of the bottleneck [13]. Taking advantage of using *Oct4ΔPE*-EGFP mice heteroplasmic for two mtDNA sequence variants, Wai et al. [14] measured both mtDNA copy number and heteroplasmy in single germ cells. They detected that PGCs possessed a mean of ∼280 mtDNA copies (median 145) per cell at 8.5 dpc, the earliest stage at which PGCs could be isolated in their mouse strain, and a mean of ∼2000–6000 copies per germ cell between 9.5 dpc and 16.5 dpc. Interestingly, despite the remarkable low mtDNA content in 8.5 dpc PGCs, an increase in genotypic variance at any point during embryonic oogenesis was not found. Instead an inequality of genotypic variance in germ cells between postnatal day 8 and day 11 was discovered. It was concluded that the mitochondrial genetic bottleneck occurs not during embryonic oogenesis but during postnatal oocyte maturation through replicating a subpopulation of genomes [14]. The two reports by Cao et al. [12] and Wai et al. [14], therefore, reached concordant conclusions that the genetic bottleneck is not attributed to the decline in PGC mtDNA content. However, it remains unknown why three studies aimed to determine mtDNA copy number in single PGCs produced distinct results at early developmental stages, that is, while Cao et al. [12] detected no severe decrease of mtDNA copies in PGCs, the other two studies found a significant reduction in PGC mtDNA content at 7.5 and 8.5 dpc, respectively [13],[14]. One proposed explanation is that the ALP histochemical staining used for PGC isolation may confound mtDNA estimation made by qrt-PCR, while the PGC-specific markers, *Stella*-GFP and *Oct4ΔPE*-EGFP, omit the staining step and may interfere less with qrt-PCR amplification [13],[14]. The other possible explanation is that the isolation of PGCs using flow cytometry may introduce PGC sample contamination [14],[15]. Given the important implications of a mitochondrial bottleneck in mtDNA disease inheritance, it is vital to determine the true germline mtDNA copy number in order to fully understand the underlying mechanisms of the bottleneck. In this study we manually isolated PGCs from mice expressing fluorescent proteins specifically in PGCs to by-pass ALP histochemistry and flow cytometry sorting procedures, and examined mtDNA copy number in single PGCs at different embryonic development stages. The present results confirm that no severe reduction of mtDNA content occurs in PGCs.

## Results/Discussion

In this study we have focused on our reassessment of mtDNA copy number in PGCs at 7.5 and 13.5 dpc. These two stages were chosen because 7.5 dpc was the only stage showing significantly different PGC mtDNA copies between the studies using either ALP staining or PGC-specific reporter transgenic mice for PGC identification, and the mean mtDNA copy numbers detected in PGCs at other stages did not differ very much [12],[13]. PGCs of 13.5 dpc would serve to compare the mtDNA copy numbers in PGCs between the two developmental stages and between genders. 7.5 and 13.5 dpc PGCs were isolated from mice expressing: i) mRFP protein under the control of a *Blimp1* genomic fragment [16]; and ii) GFP protein under the control of an *Oct4* genomic fragment with deletion of the proximal enhancer, respectively [17],[18]. *Blimp1*-mRFP and *Oct4ΔPE*-GFP are reliable PGC markers at the corresponding developmental stages [18],[19]. Our construct of *Oct4ΔPE*-GFP is identical to that in Yoshimizu et al. [18].

Due to subtle variations in the time of conception, there is a variation in the developmental stage of individual mouse embryos at any given time point [20]. To examine whether PGC mtDNA copy number differs among 7.5 dpc embryos of different developmental stages we have identified and divided 7.5 dpc embryos into two groups having morphology of early bud (EB) stage and late bud (LB) stage, respectively.

### Validation of *Blimp1*-mRFP as a reliable marker to identify PGCs at EB and LB stages of 7.5 dpc embryos

**Figure 1:**
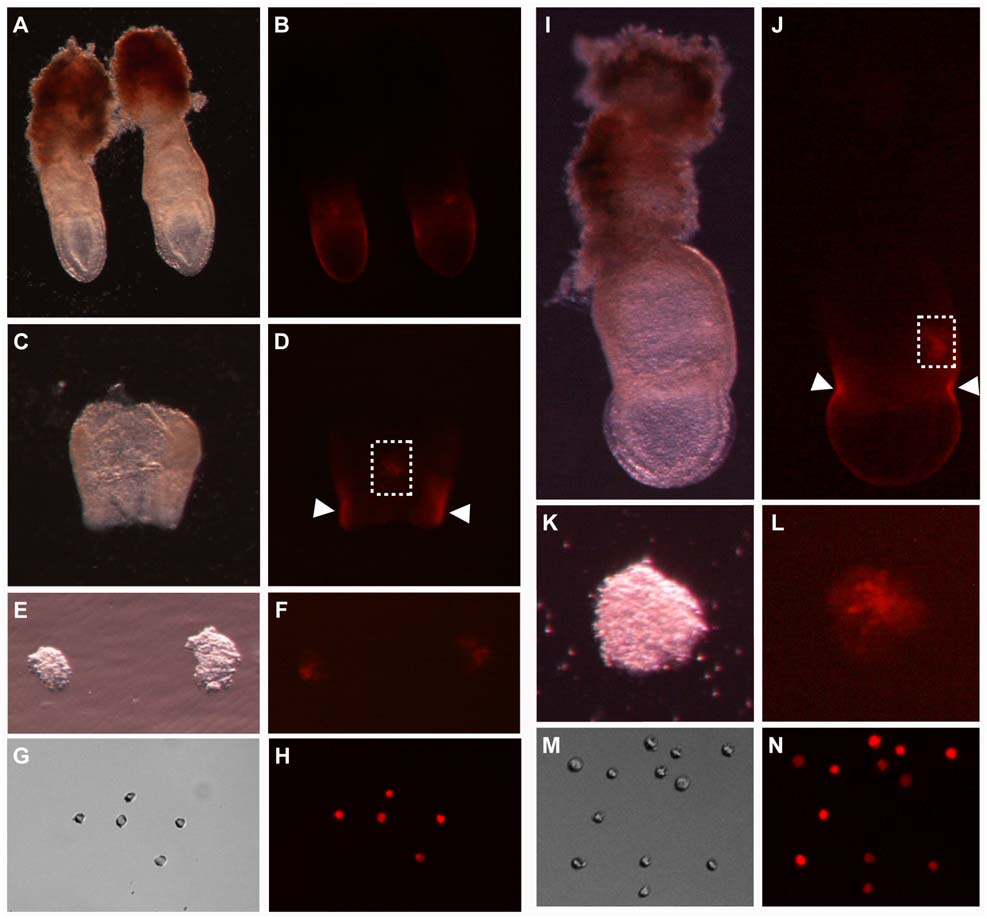
Isolation of single PGCs from 7.5 dpc early bud (A–H) and late bud (I–N) stage *Blimp1*-mRFP embryos. (A,C,E,G,I,K,M) Light microscopy images. (B,D,F,H,J,L,N) Fluorescent microscopy images. At both EB and LB stages mRFP was detected as a cluster in the posterior part of the embryo (dashed rectangle) and in the visceral endoderm (arrowheads). For isolating PGCs, the posterior regions bearing PGCs were cut off carefully to remove the visceral endoderm (E,F,K,L). The resulting tissue fragments were disaggregated through trypsinization, and single PGCs with fluorescence were collected unambiguously using micromanipulators under a fluorescent microscope (G,H,M,N).

**Figure 2:**
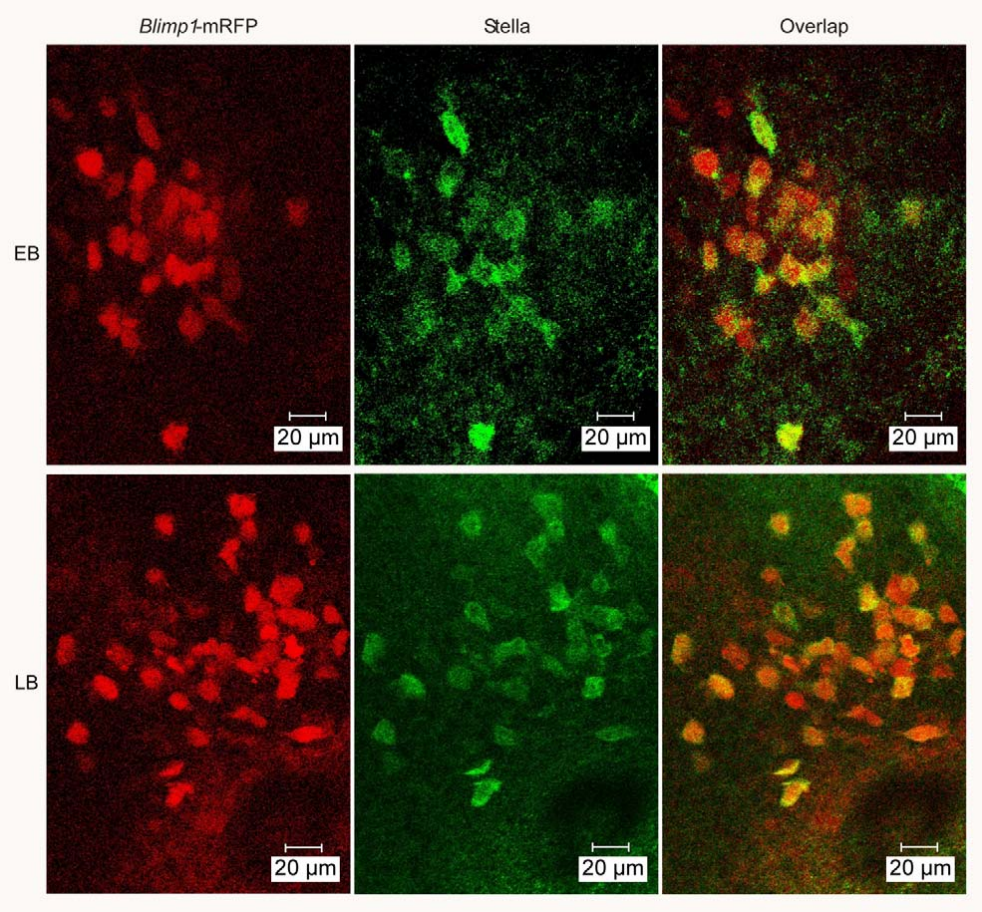
Stella expression in the posterior region of *Blimp1*-mRFP transgenic mouse embryos. *Blimp1*-mRFP (red, left column), Stella (green, middle column), and overlapped images (right column) at the EB (top row) and LB (bottom row) stages.

**Figure 3:**
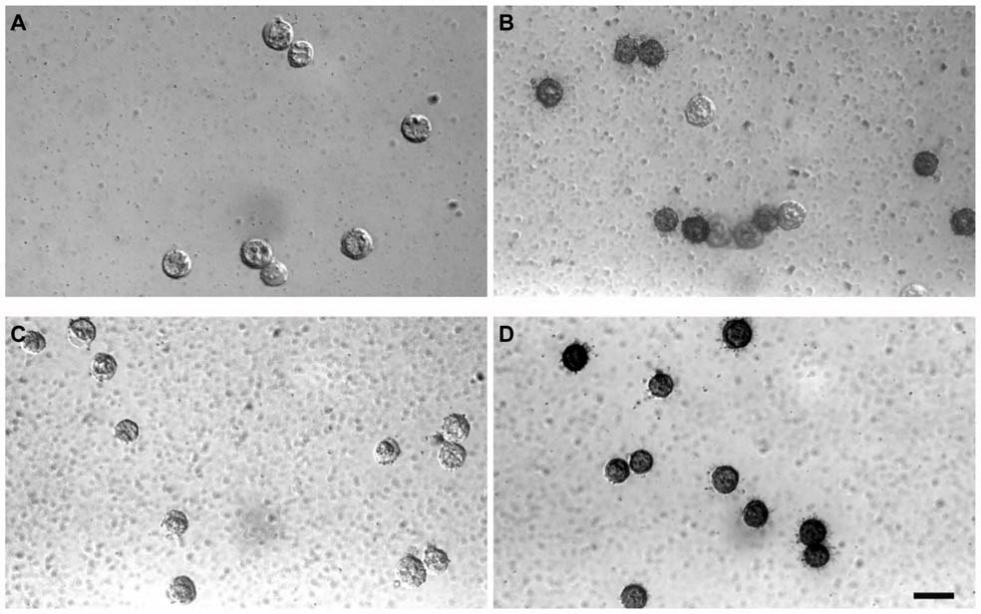
Alkaline phosphatase staining pattern of cells from the posterior region of *Blimp1*-mRFP embryos. (A,B) Cells from EB stage. (C,D) Cells from LB stage. (A,C) *Blimp1*-mRFP negative cells. (B,D) *Blimp1*-mRFP positive cells. Scale bar, 20 µm.

*Blimp1* expression marks nascent PGCs as well as precursors of PGCs in early developing mouse embryos. In the restricted posterior region (after removal of visceral endoderm) of 7.5 dpc embryos, *Blimp1* has been proved to express specifically in PGCs [19]. To facilitate the isolation of PGCs without any staining steps at 7.5 dpc, we used bacterial artificial chromosome (BAC) transgenic mice in which monomeric red fluorescent protein gene (mRFP) was inserted into the *Blimp1* locus [16]. First we determined the *Blimp1*-mRFP expression profile. At both EB and LB stages mRFP expression was observed at the posterior end of the embryonic ectoderm and visceral endoderm in embryos (Figure 1B and 1J), consistent with the endogenous *Blimp1* expression [19]. To further characterize *Blimp1*-mRFP positive cells, embryos were immuostained for Stella (PGC7), a PGC-specific marker, with the anti-Stella antibody whose specificity for PGCs has been proved [21]. Stella was detected exclusively in PGCs located at the posterior region of the embryos. All Stella positive cells were *Blimp1*-mRFP positive. At EB and LB stages Stella protein was expressed in 62.5% and 90% of *Blimp1*-mRFP positive cells in the posterior region, respectively (embryo number = 2 at both stages) (Figure 2), which is comparable with the results of Seki et al. [22]. As a second assay, we stained cells isolated from the posterior fragments (visceral endoderm removed) of *Blimp1*-mRFP embryos for alkaline phosphatase, another classical marker of PGCs. Cells were divided into *Blimp1*-mRFP positive and negative two groups prior to staining. At the EB stage, *Blimp1*-mRFP positive and negative cells were 79.4% (n = 34) and 0% (n = 28) positive for ALP staining, while at the LB stage, 92.9% (n = 28) and 0% (n = 40), respectively (Figure 3). The results agree with that *Blimp1* expression precedes that of Stella and alkaline phosphatase [19]. The cells positive for *Blimp1*-mRFP but negative for Stella are either precursors of PGC or PGCs with weak Stella expression beyond the detection limit of anti-Stella antibody. Taken together, the *Blimp1*-mRFP expression profile of our *Blimp1*-mRFP line is highly similar to that of *Blimp1* transgenic mice reported [19],[22]. Our *Blimp1*-mRFP, therefore, can be used as a reliable marker to identify PGCs in the posterior region of the embryo at EB and LB stages.

### Moderate copy number (mean >1,000) of mtDNA in PGCs and low copy number of mtDNA in embryonic somatic cells

**Figure 4:**
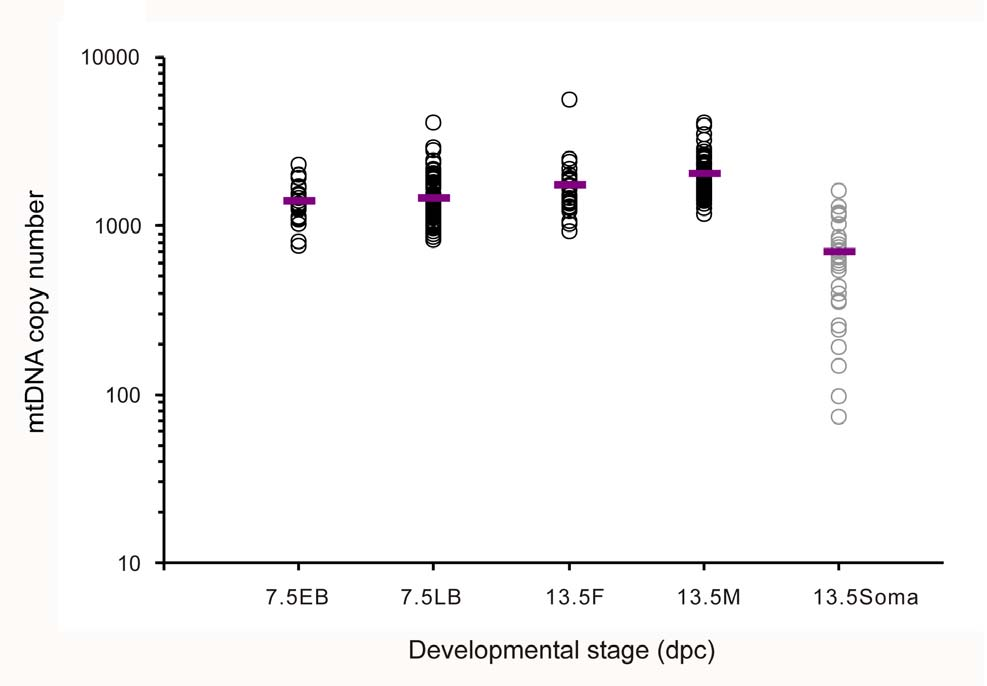
mtDNA copy number in single cells of 7.5 and 13.5 dpc mouse embryos (logarithmic scale). Open black circles, primordial germ cells (PGCs); Open grey circles, somatic cells. Each circle represents a single cell. Horizontal lines indicate mean values. EB, early bud stage. LB, late bud stage. F, M, and Soma denote samples from female PGCs, male PGCs, and gonadal somatic cells, respectively.

The mean numbers of mtDNA molecules in single 7.5 dpc EB and LB, 13.5 dpc female and 13.5 dpc male PGCs were 1396, 1479, 1747 and 2039, respectively. Of note, no extremely low mtDNA copy number was detected in any PGCs, and the minimum number of mtDNA copies determined in single PGCs was 767. The variation in mtDNA copy number per cell was similar for each group of PGCs (CV = 0.25–0.45, Table 1, Figure 4). At 13.5 dpc no significant difference in the average mtDNA copy number between female and male PGCs was observed (*t*-test, P = 0. 06). In contrast, somatic cells from the gonad were found to contain less than half the number of mtDNA copies found in PGCs at 13.5 dpc (mean mtDNA copy number in somatic cells = 702) (Table 1, Figure 4). All these results are, therefore, in very good agreement with previous estimates obtained from PGCs identified by alkaline phosphatase activity [12], taking into account that the cell samples in the present study were collected randomly without cell size classification. The present data confirm our previous findings [12]: i) There is no occurrence of remarkable reduction of mtDNA copies in early PGCs; ii) The amount of mtDNA molecules in PGCs is moderate (mean >1000 copies, comparable with that in adult somatic cells [23]) and consistent across stages; and iii) Embryonic somatic cells possess much lower mtDNA amount than PGCs.

**Table 1:** mtDNA copy number in single cells of early developing mouse embryos.

Stage	Sorting marker	Gender	Cell size	n	Mean mtDNA copy number (×10^2^)	Median mtDNA copy number (×10^2^)	Range (×10^2^)	CV
*Primordial germ cells*								
7.5 dpc	ALP staining (+)		L^a^	19	23.16	21.54	16.88–33.35	0.23
	ALP staining (+)		M^a^	19	20.01	18.37	12.44–31.18	0.29
	ALP staining (+)		S^a^	15	17.30	16.36	12.58–31.43	0.26
**EB**	***Blimp1*** **-mRFP (+)**			**25**	**13.96**	**13.34**	**7.67–23.27**	**0.25**
**LB**	***Blimp1*** **-mRFP (+)**			**83**	**14.79**	**13.86**	**8.18–40.75**	**0.35**
13.5 dpc	ALP staining (+)	Female	L^a^	15	36.56	27.49	18.78–94.50	0.65
	ALP staining (+)	Female	M^a^	25	18.43	18.16	11.44–32.04	0.25
	ALP staining (+)	Female	S^a^	31	15.32	14.04	9.95–32.35	0.32
	***Oct4ΔPE*** **-GFP (+)**	**Female**		**35**	**17.47**	**17.03**	**9.23–55.98**	**0.45**
	ALP staining (+)	Male	L^a^	16	30.53	27.61	12.9–87.28	0.56
	ALP staining (+)	Male	M^a^	28	18.95	19.60	3.78–34.52	0.33
	ALP staining (+)	Male	S^a^	26	13.50	12.88	3.26–25.06	0.37
	***Oct4ΔPE*** **-GFP (+)**	**Male**		**65**	**20.39**	**19.68**	**11.90–40.79**	**0.29**
*Somatic cells*								
13.5 dpc	ALP staining (−)	Female	L^a^	10	6.45	6.07	3.44–8.90	0.25
	ALP staining (−)	Female	S^a^	9	4.86	4.50	0.39–9.55	0.63
	***Oct4ΔPE*** **-GFP (−)**	**Female**		**35**	**7.02**	**6.54**	**0.74–16.29**	**0.58**

Range, range in mtDNA copy number of cells. CV, coefficient of variation in mtDNA copy. EB, early bud stage. LB, late bud stage. number. (+) and (−) stand for positive and negative, respectively.

a Data are from Cao et al. [12]. L, large size cell. M, medium size cell. S, small size cell. Somatic cells are from 13.5 dpc female gonads.

How can we explain the discrepant findings among Cree et al. [13], Wai et al. [14] and ours? Cree et al. [13] detected median ∼200 mtDNA copies (mean 451) in PGCs at 7.5 dpc and more than 1000 copies at later stages. This result may be associated with the aspects of their methodology for PGC sorting. Cree et al. [13] identified and sorted PGCs by flow cytometry. Isolation of PGCs using flow cytometry was shown to be inaccurate for early developmental stage embryos [14],[15]. Szabo et al. [15] carried out PGC sorting from *Oct4ΔPE*-EGFP transgenic mice that were generated using the construct identical to that in Yoshimizu et al. [18]. This *Oct4ΔPE*-EGFP were reported to be specifically expressed in PGCs from 8.5 dpc onwards [17],[18]. At 8.5 dpc (five somites) only 62% EGFP^+^ cells sorted by flow cytometry were positive for the PGC marker, whereas 96% or more were positive at 9.5 dpc and later stages [15], indicating the inaccuracy of flow cytometry in identifying GFP cells expressing a PGC marker at early developmental stages when PGCs are small in number relative to non-PGC cells in the sample. The number of PGCs in single embryos at 7.5 dpc is even smaller than at 8.5 dpc. Therefore it is possible that more than 38% of GFP^+^ cells sorted and analyzed by Cree et al. [13] at 7.5 dpc were not PGCs but somatic cells. Embryonic somatic cells have been shown to contain significantly lower amounts of mtDNA than PGCs [12],[24]. By studying whole embryos, Aiken et al. [24] reported that the mean of mtDNA copies per somatic cell was ∼300 between 6.5 dpc and 18.5 dpc, comparable with the 451 copies in the 7.5 dpc PGC sample of Cree et al. [13]. In contrast, our isolation of 7.5 dpc PGCs was performed manually under a fluorescent microscope using micromanipulators. Hence the purity of PGCs was ensured (Figure 1). Wai et al. [14] found a median of 145 mtDNA copies (mean ∼280) per PGC at 8.5 dpc and more than 1000 copies at other stages. It is currently unclear why the study of Wai et al. [14] gave the result significantly different from that of Cree et al. [13] and ours for 8.5 dpc PGCs. However, the fact that both Cree et al. [13] and Cao et al. [12] detected a mean of mtDNA copies more than 1000 per 8.5 dpc PGC weakens the possibility of extremely lower mtDNA copy number (mean ∼280) in 8.5 dpc PGCs.

Why do PGCs contain more than 1000 mtDNA copies in sharp contrast with consistent ∼300 per embryonic somatic cell across stages between 6.5 dpc and 18.5 dpc? One explanation is that the moderate mtDNA copy number in PGCs allows the cell to have an elevated tolerance for less deleterious mtDNA mutations and serves as a device to maintain the adaptive potential of mtDNA genome, which for a nuclear genome is achieved via diploidy by means of sexual reproduction. On the other hand, a tight physical bottleneck in somatic lineages during embryonic development enables pathogenic mtDNA mutations (both severe and less deleterious variants) to rapidly segregate and be eliminated more efficiently. The mtDNA copy number in gonadal somatic cells at 13.5 dpc was much higher (mean 702) than the average mtDNA level per cell of the whole embryo (mean ∼300) [24], suggesting that the physical bottleneck in embryonic somatic cells appears lineage specific which might be associated with tissue-specific cell function and bioenergic demands. Alternatively, the moderate mtDNA copy number may be entailed to meet the energetic demand for PGC migration, mitotic replication and cell function. Recent findings from two research groups have provided new insight into the mtDNA segregation between generations. Wai et al. [14] showed that the mtDNA genetic bottleneck occurs not during embryonic oogenesis but during postnatal oocyte maturation as a result of replication of a subgroup of mtDNAs. Fan et al. [25] demonstrated a strong and rapid purification selection of mtDNA in the germline, eliminating severe mutations within three generations in mice. If the purifying filter takes effect only before the cease of PGC proliferation by 13.5 dpc, females carrying severe mtDNA mutations would produce progeny with a similar frequency of mutant variants in any given litter. However the female mice harbouring either ND6 frameshift mtDNA or mtDNA with a 4696-bp deletion produced offspring with a declining proportion of mutant mtDNA in their successive litters [25],[26], indicating that the purifying filter acts after 13.5 dpc. Data from ND6 frameshift mtDNA mice have shown that the selection appeared not to operate at embryonic stage through selectively eliminating foetuses with the highest percentages of mutation [25]. One possibility is that the purifying selection acts at the cell level through a competition between germ cells with different proportions of severe mutant variants. When germ cells with the highest ratio of severe mutations are held in the ovary for a long time they might gradually lose their competence in competing to develop into mature oocytes, as opposed to those with lower ratio of mutations, due to the cumulative detrimental effect of mutation on cells over time. Consequently the proportion of severe mutant mtDNA would be lower in younger siblings and the severe mutation would progressively be lost and eventually disappear in following generations, which are the patterns revealed in the mouse pedigrees harbouring either a ND6 frameshift mutation [25] or a mtDNA large deletion mutation [26]. In human pedigrees, a decline in the mtDNA mutation load in younger siblings has not been found [27]–[30]. Pedigree studies of pathogenic mutations in human mtDNA have mainly focused on large deletion mutations and point mutations (either missense mutations in protein genes or base substitution mutations in tRNA and rRNA genes). These point mutations are apparently milder than the ND6 frameshift mutation resulting in a premature termination of gene transcription [25] and the mtDNA large deletion mutation where a number of genes were missing from the genome [26], and hence, may not undergo a strong purification selection in the germline. Deletion mtDNAs in humans were suggested not to be maternally transmitted, rather to arise spontaneously, since mothers and siblings of affected individuals rarely harboured deletion mtDNAs [31]. It is possible that the purification selection of germline mtDNA is much stronger in humans than in mice, and virtually no large deletion mtDNAs in human oocytes would survive this selection. In rare cases where a deleted genome slips through, clonal expansion of such a deleted molecule(s) in embryogenesis could lead to mitochondrial disease in the child. Taken together, these may explain the failure to find a decrease in mtDNA mutation load in offspring as maternal age increases in human pedigrees. Alternatively, severe mtDNA mutations could be selected against at the organelle level [32]. Mitochondria with a higher proportion of mutation diminish with time, leading to a decline of mutant mtDNA in oocytes. The selection at the organelle level could be oocyte-specific, since it is not supported by measurements of deletion mutation loads in non-dividing somatic tissues in which mutant mtDNAs accumulate with age [33],[34]. It is known that the number of mtDNA copies per mitochondrion decreases during oogenesis, and eventually reaches approximately a mtDNA per mitochondrion in mature oocytes [35]. This decline of mtDNA copy number in single mitochondria may render the mitochondrion more sensitive to severe mutations and facilitate the elimination of mutant mitochondria in oocytes.

In summary, our new data show solid evidence that there is no drastic reduction of mtDNA copy number in PGCs. The amount of mtDNA in single PGCs is moderate and comparable at 7.5 and 13.5 dpc stages, while somatic cells in 13.5 dpc gonads contain far fewer mtDNA copies than PGCs. The results from this and our previous study [12] are highly consistent despite using different mouse lines (wild-type mice vs. transgenic mice) and different PGC isolation methods. These results reinforce our conclusion that the mitochondrial bottleneck occurs without reduction of mtDNA copy number in germline cells, which is also supported by the study showing the mtDNA genetic bottleneck occurrence as a result of replication of a subpopulation of mtDNA genome and not a remarkable reduction in PGC mtDNA content [14]. The rapid segregation of mtDNA variants between generations is likely achieved through a genetic bottleneck resulting from replication of a subgroup of mitochondrial genome during oocyte maturation [14] in combination with a strong purifying selection against severe mutations over a long time period in the germ line [25]. The reconfirmation of mtDNA copy numbers in PGCs in the present study is of importance because it clarifies the confusions and the information of germline mtDNA content will facilitate the development of therapeutic strategies blocking the mitochondrial disease transmission from mother to progeny.

## Materials and Methods

### Transgenic mouse strains

*Blimp1*-mRFP transgenic mice (BDF1 background) were generated using a 203-kb bacterial artificial chromosome (BAC) expressing mRFP under the control of *Blimp1* regulatory elements as previously described [16]. *Oct4ΔPE*-GFP mice (BDF1 background) were generated using the construct identical to that of Yoshimizu et al.'s [18], carrying the GFP sequence driven by an 18-kb *Oct4* genomic fragment with deletion of the proximal enhancer [16].

The *Blimp1*-mRFP mouse strain (RBRC01830) and *Oct4ΔPE*-GFP mouse strain (RBRC00821) were provided by the RIKEN BioResource Center (http://www.brc.riken.jp/inf/en/). All animal experiments were approved by the Institutional Animal Experiment Committee of RIKEN BioResource Center and of the Tokyo Metropolitan Institute of Medical Science.

### Immunostaining analyses

7.5 days post coitum (dpc) embryos of *Blimp1*-mRFP mice were collected in Dulbecco's Modified Eagle's Medium (DMEM) (Invitrogen) supplemented with 10% FCS, and were further precisely classified as at either early bud (EB) or late bud (LB) stage according to the morphological landmarks [20]. Immunohistochemistry assay was carried out as in Sugimoto and Abe [16]. Briefly, isolated embryos were incubated in PBS containing 0.5% Triton X-100 for 3–5 min on ice and fixed with 4% paraformaldehyde in PBS for 10 min at room temperature. Following stringent washing, the embryos were incubated with anti-Stella primary antibody (1∶1,000) (kindly provided by Dr. Toru Nakano) diluted with blocking buffer (PBS containing 1% BSA and 0.1% Triton X-100) for 1 h at room temperature, washed with washing buffer (PBS in 0.1% Triton X-100), and incubated with Alexa-Fluor-488 conjugated goat-anti-rabbit secondary antibody diluted in blocking buffer for 45 min at room temperature. Fluorescent images were acquired in Z series every 0.82 µm using Zeiss LSM510 Meta confocual microscope.

Posterior fragments after removal of visceral endoderm from *Blimp1*-mRFP embryos of EB and LB stages were dissected out. The fragments were disaggregated with Trypsin. Cells were divided into *Blimp1*-mRFP negative and positive groups, and underwent alkaline phosphatase (ALP) staining as in Cao et al. [12].

### Isolation of single PGCs and single somatic cells

(C57BL/6N×DBA/2N)F1 female mice were mated with *Blimp1*-mRFP and *Oct4ΔPE*-GFP males. 7.5 and 13.5 dpc embryos were collected in DMEM supplemented with 10% FCS. The restricted posterior parts of 7.5 dpc EB and LB *Blimp1*-mRFP embryos bearing PGCs (visceral endoderm removed, Figure 1) and the gonads of 13.5 dpc *Oct4ΔPE*-GFP embryos were isolated using fine needles, and incubated in Trypsin-EDTA solution (Sigma) at 37°C for 10 min. Cells were dissociated in DMEM supplemented with 10% FCS by pipetting, were pelleted and washed twice in PBS and were resuspended in modified Dulbecco's phosphate-buffered medium (PBI). Single PGCs expressing fluorescent proteins and the gonad somatic cells were randomly collected under a fluorescent microscope using micromanipulators (Leica). PGCs from female and male gonads were isolated separately at 13.5 dpc, at which time the gender of the fetus can be identified microscopically.

### Estimation of mtDNA copy number by quantitative real-time PCR (qrt–PCR)

Single cells were extracted and used directly for qrt-PCR analysis as previously described [12],[36]. Quantification of absolute mtDNA copy number per cell was carried out by the standard curve method. Details of the probe, primers and standard DNA are as in Cao et al. [12]. To evaluate whether pure DNA of plasmids containing mouse mtDNA fragment is suitable to serve as standard DNA for the measurement of mtDNA copy number in single cells, we performed qrt-PCR on samples containing the plasmid DNA in the presence of a single mtDNA-less ρ^0^B82 cell using pure plasmid DNA as standard DNA as described previously [12], and compared the sample plasmid copy numbers estimated by PCR with their corresponding true copy numbers. ρ^0^B82 line was derived from L cells [37],[38] whose mtDNAs encompass the same mtDNA fragment integrated into the plasmid. Mixing the plasmid DNA with a ρ^0^B82 cell creates sample conditions closer to that of single cell DNA for PCR amplification. It showed that the estimated copy values highly correlated with their true copy values (r^2^>0.99) over a range of mtDNA concentrations that comprise the values detected in our single cell studies (Figure S1), indicating that a systematic underestimation or overestimation of actual plasmid copies did not occur. Therefore the pure plasmid DNA is suitable for using as standard DNA for our determination of mtDNA copies in single cells. This qrt-PCR system could detect as few as 10 copies of standard DNA template reliably. The linear regression analysis of all standard curves for samples with copy numbers between 10 and 10^6^ showed a high correlation (r^2^>0.99). Samples from both 7.5 and 13.5 dpc were measured in at least duplicate plates to avoid systematic errors that may be caused by transfer of standard DNA or other factors. The means of mtDNA copy number determined from the plates were assessed by *t*-test. No significant differences were found between the plates carrying the samples of matched categories.

## Supporting Information

Figure S1 Correlation between plasmid copy values measured by qrt-PCR and their corresponding true copy values in samples containing plasmid DNA in the presence of a ρ^0^B82 cell. Plasmid DNA was quantified and serially diluted. Each dilution was added with a single ρ^0^B82 cell and underwent qrt-PCR amplification in triplicate using PCR conditions referred to in the manuscript text. The graphs represent the results of two independent assays. A tight correlation between the measured copy number values and their true copy number values was found in both assays (R^2^>0.99).
(8.43 MB TIF)
